# Suture-loop anchoring at the distal end of a pancreatic stent: a novel approach to prevent proximal migration

**DOI:** 10.1055/a-2625-3711

**Published:** 2025-07-14

**Authors:** Mingwen Guo, Wenguang Yang, Ping Wang, Yuhong Ren, Bin Yang, Sichao Wen, Danqing Liu

**Affiliations:** 1 Department of Gastroenterology, Qionglai Medical Center Hospital, Qionglai, China; 2 388288 Department of Hepatobiliary Surgery, Third Military Medical University Southwest Hospital, Chongqing, China

Proximal migration of pancreatic stents, though rare (0.2%) 1 , remains a serious complication of endoscopic retrograde cholangiopancreatography (ERCP) that requires complex retrieval methods including endoscopic grasping, pancreatoscopy, or surgery 2 . Recent advances include endoscopic ultrasound (EUS)-guided retrieval 3 , yet these approaches demand expertise and resources.

We propose an innovative preventive strategy. An absorbable suture is threaded through the terminal side hole, and the length of the suture after knotting is kept at approximately 3 cm ( Fig. 1 **a** ). The video documents a 78-year-old jaundiced patient in whom a trainee inadvertently deployed a 5-Fr stent deeply into the pancreatic duct ( Fig. 1 **b** ). The supervising physician grasped the externalized suture with rat tooth forceps ( Fig. 1 **c** , Video 1 ), successfully repositioning the stent ( Fig. 1 **d** ) and enabling subsequent biliary stenting. This suture-loop technique provides a simple yet reliable solution for preventing pancreatic stent migration. The technique is particularly suitable for trainees and is recommended for widespread clinical adoption.

**Fig. 1:**
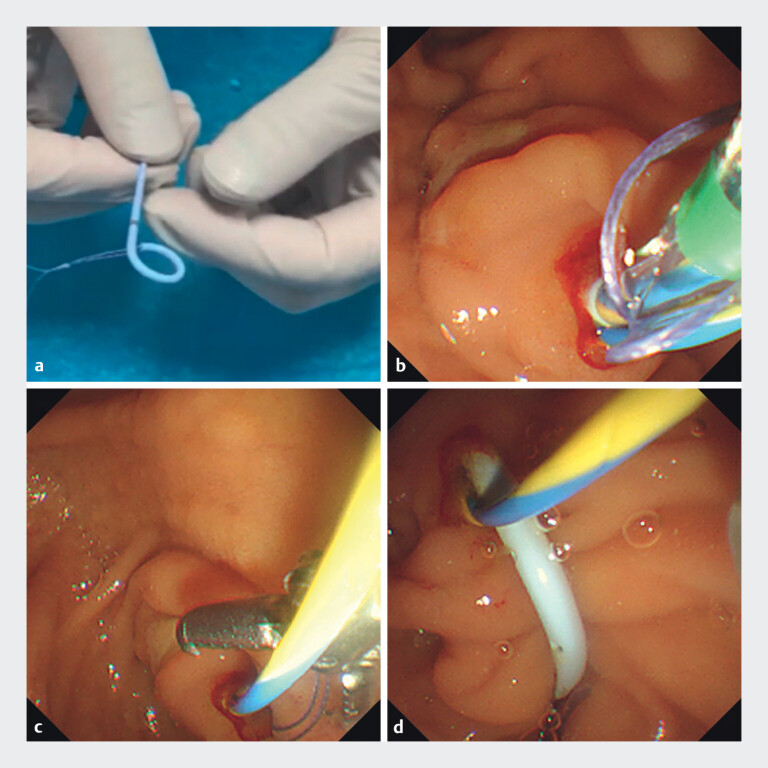
Novel suture-loop anchoring at the distal pancreatic stent: Technique demonstration and migration rescue. **a** Suture-loop anchoring technique: An absorbable suture is threaded through the distal side hole of the pancreatic stent, with the knotted suture length maintained at 3 cm. **b** Deep migration of a 5-Fr pancreatic stent during trainee-led deployment in a jaundiced patient. **c** Retrieval of the migrated stent via the externalized suture-loop using a rat tooth forceps. **d** Repositioned pancreatic stent enabling subsequent biliary intervention.

Demonstration of suture-loop technique for pancreatic stent fixation via externalized suture retrieval, enabling migration rescue.Video 1

Endoscopy_UCTN_Code_TTT_1AR_2AZ

## Notes

Endoscopy E-Videos https://eref.thieme.de/e-videos
*E-Videos* is an open access online section of the journal *Endoscopy* , reporting on interesting cases and new techniques in gastroenterological endoscopy. All papers include a high-quality video and are published with a Creative Commons CC-BY license. Endoscopy E-Videos qualify for HINARI discounts and waivers and eligibility is automatically checked during the submission process. We grant 100% waivers to articles whose corresponding authors are based in Group A countries and 50% waivers to those who are based in Group B countries as classified by Research4Life (see: https://www.research4life.org/access/eligibility/ ). This section has its own submission website at https://mc.manuscriptcentral.com/e-videos .
